# Exploring the Shared Pathogenesis Mechanisms of Endometriosis and Cancer: Stemness and Targeted Treatments of Its Molecular Pathways—A Narrative Review

**DOI:** 10.3390/ijms252312749

**Published:** 2024-11-27

**Authors:** Melinda-Ildiko Mitranovici, Dan Costachescu, Septimiu Voidazan, Mihai Munteanu, Corneliu-Florin Buicu, Ioan Emilian Oală, Viviana Ivan, Adrian Apostol, Ioana M. Melinte, Andrada Crisan, Lucian Pușcașiu, Romeo Micu

**Affiliations:** 1 Department of Obstetrics and Gynecology, Emergency County Hospital Hunedoara, 14 Victoriei Street, 331057 Hunedoara, Romania; oalaioanemilian@gmail.com; 2 Department of Orthopedics-Traumatology, Urology, Radiology and Medical Imaging, University of Medicine and Pharmacy Victor Babes, 2 Eftimie Murgu Square, 300041 Timisoara, Romania; 3 Department of Epidemiology, “George Emil Palade” University of Medicine, Pharmacy, Sciences and Technology, 540142 Targu Mures, Romania; septimiu.voidazan@umfst.ro (S.V.); florin_buicu@yahoo.com (C.-F.B.); ioanammelinte@gmail.com (I.M.M.); crisanandrada@yahoo.com (A.C.); puscasiu@gmail.com (L.P.); 4 Faculty of Electrical Engineering, Technical University, George Baritiu Street, 400394 Cluj-Napoca, Romania; mihai.munteanu@ethm.utcluj.ro; 5 Department VII, Internal Medicine II, Discipline of Cardiology, University of Medicine and Pharmacy Victor Babes, 2 Eftimie Murgu Square, 300041 Timisoara, Romania; ivan.viviana@umft.ro (V.I.); adrian.apostol@umft.ro (A.A.); 6 Department of Obstetrics and Gynecology, University of Medicine and Pharmacy Iuliu Hatieganu, 400347 Cluj-Napoca, Romania; romeomicu@hotmail.com

**Keywords:** endometriosis, cancers, stem cells, stemness, molecular pathways

## Abstract

Endometriosis is a benign disease but with malignant behavior, sharing numerous features with cancers. Endometriosis is the development of endometrial tissue outside the uterus, with the presence of both glands and stroma. Approximately 10% of women of reproductive age suffer from endometriosis; it involves high social costs and affects the patient’s quality of life. In this review, we attempt to capture the pathogenesis mechanisms that are common to endometriosis and cancer based on molecular biology, focusing more on the principle of immunological changes and stemness. Clinical applicability will consist of targeted treatments that represent future directions in these diseases, which impose a burden on the healthcare system. Unlike endometriosis, cancer is a disease with fatal evolution, with conventional treatment based on chemo/radiotherapy. Here, we focus on the niche of personalized treatments that target molecular pathways. Our findings show that, in both pathologies, the resistance to treatments is due to the stemness of the stem cells, which might play a role in the appearance and evolution of both diseases. More research is needed before we can draw firm conclusions.

## 1. Introduction

Endometriosis is the development of endometrial tissue outside the uterus, with the presence of both glands and stroma [1,2,3]. The first description of ectopic endometrium dates back to 1860 [4]. Endometriosis is a benign disease with malignant behavior [2]; 10% of women of reproductive age suffer from endometriosis [1,5,6,7,8]. The most common symptoms are infertility and chronic pelvic pain [1,2,5]. The estimated prevalence is 35–50% of the symptomatic population [5]. Even with treatment, residual symptoms remain present [1], affecting the quality of life and increasing the need for surgical interventions and assisted reproduction methods to improve fertility [1,6,9].

Endometriosis affects women of reproductive age, most frequently between 30 and 40 years old [10]. An accurate diagnosis requires surgical intervention associated with biopsy [11,12]. Endometriosis is more frequent in the Asian population [13,14,15].

The association of endometriosis with cancers is well known and studied. The incidence of endometriosis in patients with ovarian cancer is 8–30% [16]. Studies on the Swedish population have shown that the risk of malignancy in the presence of endometriosis is increased by 4.2-fold. These types of cancers are lower-grade, predominantly endometrioid and clear-cell [16]. In addition, there is an increased risk of extraovarian cancers (e.g., breast cancers and non-Hodgkin lymphoma) in women with endometriosis [13,14,15].

Endometriosis can also be associated with autoimmune diseases and chronic fatigue syndrome [1,2,5,17]. Due to the fact that the patients develop vascularization and innervation disorders, it is a systemic disease rather than a localized one, with multiple complications, affecting the patient’s quality of life [5]. Endometriosis involves high social costs and poses a critical social problem because it negatively affects women’s daily lives, including work, sleep, emotions, social interactions, sex life, finances, and fertility [1,9,18]. Unfortunately, there is no definitive cure; therefore, it is necessary to avoid contracting this puzzling disease and to spare women from the difficulties of treating it [19].

To date, the most accepted hypothesis is from Sampson [5,14] which includes the spread of endometrial cells via retrograde menstruation. Nevertheless, not all women with menstrual reflux develop endometriosis; they may have a better immune system or a better peritoneal barrier than women with endometriosis [5,14,19]. A laparoscopy with histological verification remains the most important diagnostic procedure, although it is still not free of the risk of morbidity or even mortality. As non-invasive methods are not reliable for the diagnosis of endometriosis, the average time between the onset of symptoms and the diagnosis ranges between 8 and 10 years [19]. This is the reason for the involvement of so many international organizations and the granting of so much funding for the study of this pathology. Management, classification, and finding biomarkers that facilitate diagnosis and targeted, individualized treatment are continuous concerns in modern medicine [20,21]. The course of progression of endometriosis is still not fully understood. While in some studies the disease is progressive, in others, stagnation or even regression is mentioned [19]. As the gold standard in diagnosis remains laparoscopy, as mentioned above, with lesion visualization and anatomopathological examination, biomarkers are sought from accessible fluids such as blood, urine, and peritoneal fluid, which are useful in diagnosis and can avoid the need for laparoscopy, an expensive procedure with associated risks [2,5,22].

Predisposing factors for endometriosis could include low birth weight, exposure to high levels of diethylstilbestrol-type estrogen in utero, menarche at a young age, short timespans between periods, and low BMI. At the same time, childbirth and breastfeeding, a diet high in antioxidants, and physical exercise play protective roles against the disease [2]. Environmental risk factors in the development of this pathology include toxins, alcohol consumption, smoking, and excessive meat consumption. High-fat and sugar-rich diets can promote carcinogenesis from endometriotic lesions [23]. However, we cannot draw definitive conclusions about lifestyle, because there have been no extensive studies in this regard [2,24,25].

Different factors involved in migration, proliferation, angiogenesis, and adhesion have been sought through histopathological examination to serve as biomarkers [5]. Single tests or a combination of several biomarkers can be used. Angiogenic factors, growth factors, markers of apoptosis and DNA repair, cell adhesion molecules, hormonal markers, matrix-related molecules (e.g., osteopontin), immune system and inflammatory markers, inflammasome NLRP3 (nod, LR and pyrin domain protein 3), oxidative stress markers, microRNAs, microDNAs, tumor markers, and proteomics have been studied, with molecular biology representing the future of this research field [5,25,26,27,28,29]. An association between endometriosis and some autoimmune diseases, such as systemic lupus erythematosus, has also been found, and common biomarkers involved in the complement and coagulation cascade have been studied, but without much success [2,5,6,9,22,30]. The most studied was CA125, a glycoprotein expressed on coelomic epithelial tissues, with limited value [5]. The most complex review in this regard was carried out by the Cochrane Database, and one of the reasons for the failure to establish the usefulness of biomarkers was the low methodological quality of the studies. A questionnaire with clinical parameters showed a very good prediction of endometriosis and could be used as a non-invasive tool [31]. More accurate studies are needed, with a methodology that can be reproduced.

Disease management consists of surgery, hormonal therapy, and individualized pain relief [2,5,6,17,20,21]. Hormonal therapy consists of combined contraceptives, weak androgens, and GnRH (gonadotropin-releasing hormone) agonists and antagonists; these relieve the symptoms but are accompanied by numerous adverse effects, including the suppression of menstruation, breast discomfort, irritability, and bone loss [5,20,21]. In addition, there is the possibility of resistance to hormonal therapy [3]. An explanation for therapeutic resistance, beyond the alteration of the hormonal receptors, could be that the treatment only addresses the differentiated cells, not the stem cells that propagate and spread the disease [25]. For endometriosis adhesions, dexpanthenol, injected intraperitoneally, is being studied as a scavenger of ROS (reactive oxygen species) in oxidative stress [32]. When empirical treatment fails, laparoscopy is used, which has diagnostic and therapeutic value [5]. Women from rural areas or with lower economic status do not benefit from this treatment [5]. Surgical excision reduces the pain [5,11,21]. A rigorous methodological design is necessary to minimize potential inaccuracies [6]. The solution to infertility consists of various ARTs (assisted reproduction techniques), but the most useful seems to be oocyte vitrification, and the possibility of reducing antral follicles through aggressive surgical interventions must be taken into account [3,5].

It should be noted that women with endometriosis also have common risk factors for cancers, including oxidative stress, genetics, environmental factors, and inflammation [13,14,15,16,33,34,35].

The aim of this study was to search the literature for various pathogenesis mechanisms that are common to endometriosis and cancer in general based on molecular biology, focusing more on the principle of stemness. Clinical applicability will consist of targeted treatments that represent future directions in these diseases. The present study, based on the PRISMA guidelines for conducting reviews, used a robust search strategy, utilizing electronic databases such as PubMed and Google Scholar [36].

## 2. Pathogenesis Hypotheses

- Various hypotheses have been proposed, the only generally accepted one being that of menstrual reflux; we present them below.
- The first theory, proposed by Sampson in 1927, is an implantation hypothesis which also includes dissemination through blood and lymph system [1,2,5,9,14]. The endometrial-like tissue must adhere, implant, and differentiate while avoiding the immune system. This is demonstrated by the presence of large amounts of adhesion molecules such as VCAM (vascular cell adhesion molecule), ICAM (intracellular adhesion molecule), and metalloproteinases, with mechanical transplantation [2,22].
- Müllerian transformation: Remnant Müllerian embryonic rest cells must be present at the ectopic site and are induced to differentiate into functioning endometrial cells under certain influences [1,2,18,37]. The theory of embryonic remnants’ induction would explain instances of endometriosis found in men [38,39]. Also, endometriosis can occur in women with Mayer–Rokitansky–Küster–Hauser syndrome with uterus remnants or Müllerian embryonic rests [40,41].
- Via the lymphatic or blood vessels, the unusual occurrence of lesions in other places, e.g., the lungs, brain, eyes, pleura, and nerve tissue, explained by Sampson’s theory [2,18,42]. The cells must be able to migrate, implant, and differentiate into endometrial-like tissue and evade the immune system [1,2,3,5,17,22]. Cells derived from endometriosis with stem-cell-like properties may also enter the blood circulation to migrate and implant into distant tissues [43].
- Metaplasia theory: This hypothesis involves spontaneous transdifferentiation of coelomic tissue into endometrial-like tissue at the site of the lesion under unknown exogenous influences [18,37,40].
- Genomic and proteomic abnormalities [3,24,44].
- Stem cells influenced by local immune reactions, genetic influences, and microenvironmental factors [3,18,25,35,37].
- According to other authors, deep infiltrating endometriosis (DIE) is histologically characterized by well-differentiated glandular cells and stromal cells, mixed differentiated cells, and pure undifferentiated glandular cells. These undifferentiated cells from the endometriotic lesions result from cell resistance to the suppressive effects of the immune system in the peritoneal cavity; this process allows these endometrial undifferentiated cells to infiltrate more deeply. Therefore, deep endometriosis occurs due to different pathogenetic pathways, including inflammation, neo-angiogenesis, and differentiation of undifferentiated cells, in order to adapt better and to proliferate in inhospitable anatomical sites, which is consistent with Sampson’s theory [45]. Although some authors have claimed that this hypothesis cannot explain the presence of endometriotic lesions in men subjected to hormonal treatment for prostate or bladder cancer, it has not yet been clarified whether these men are pure XY genotypes or possibly XXY chimeras or also have embryonic remnants. Similarly, endometriosis after hysterectomy was suggested to disprove the theory of Sampson; however, it can be explained by, for example, lymphatic blood spread of unremoved ectopic endometrial lesions to other locations [23,38,46,47,48]. Some authors have claimed that endometriosis is not similar to eutopic endometrium and differs in many ways such as histologic and morphologic characteristics, origin of clonality, protein expression, hormone response, or enzymatic activity, and is thus not an autotransplant. However, ectopic endometrium consists of endometrial glands and endometrial stroma and is thus histologically very similar to eutopic endometrium [49,50]. Even so, we agree in our review with Sampson’s theory, and as such we added it as the first and most accepted theory, with other authors emphasizing the importance of immunohistochemistry in demonstrating the presence of estrogen receptor (ER) and progesterone receptor (PR) in the ectopic lesions, very similar with those in eutopic endometrium [42,50].
- The connection of endometriosis and endometriosis-associated ovarian cancers with the presence of stem cells in endometriotic lesions raises questions about the possible involvement of endometrial stem cells in the carcinogenesis of some endometriosis-associated ovarian cancers [23]. Different biomarkers were sought, and the researchers found that the concentration of soluble CD44 in the sera and endometrial fluid of endometriosis patients was higher than that of healthy women. Furthermore, similar disturbances in galectins or miRNA have been observed in endometrial tissue and ovarian or endometrial cancer [23,51]. Endometriosis development involves endometrial stem/progenitor cells, a notion compatible with Sampson’s retrograde menstruation theory and supported by the demonstration of eMSCs in menstrual blood, even if there is no evidence that eMSCs are responsible for the de novo formation of ectopic endometrial glands/stroma. Evidence of cancer stem cells (CSC) in endometrial cancer indicates that new avenues for developing therapeutic options targeting CSC may become available [52].

The presence of the epithelium in endometrial lesions was explained by coloniality from a single cell with stem cell properties, including self-renewal and multi-lineage cell differentiation [2]. It is now proposed that stem/progenitor cells of at least two different origins are involved in the development of endometriosis. This initiating stem cell was thought to originate from the uterine endometrium, bone marrow translocated through fallopian tubes during menstruation, or vasculo-lymphatic routes [53]. Three main types of endometrial adult stem cells have been found so far: stromal, epithelial progenitor, and endothelial stem cells. Moreover, populations of stem cells derived from bone marrow are involved as key factors of the endometrial stem cell niche; however, there is no sufficient evidence as to how these cells are implicated in the process [54,55]. The adult unipotent stem cells can differentiate in one way, endometrial stem cells; even if they enter the blood flow, they can only differentiate in endometrial-like tissue, which might also explain the evolution of endometriosis in different organs such as the lungs, umbillicum, and brain, in addition to the implantation hypothesis of Sampson [14,55]

Epithelial–mesenchymal transition through loss of E-cadherin can be involved in the development of endometriosis [2,26,56]. The loss of polarization and cell-to-cell contact in the epithelium and the acquisition of mesenchymal cell features such as high motility are thought to be among the most important transformations in order to establish endometriotic lesions. Two stimulating signals, estrogen and hypoxia, can activate the EMT process in endometriosis [57]. Neovascularization through angiogenesis is involved, as demonstrated by the increased presence of VEGF (vascular endothelial growth factor) in the peritoneal fluid (peritoneal washing), associated with pelvic pain [2,26]. Endometriosis is a complex disease; the interaction between local cellular signaling and the microenvironment, the circulating elements, the levels of hormones and the response of receptors to them, and the oxidative stress through ROS free oxygen radicals, which might result in EMT, to which we can add the genetic component, demonstrate how little we know about the mechanisms of this disease [2,40]. The authors of other studies have proposed that EMT does not play a role in the initiation of endometriosis. It is possible that a partial EMT—but without the loss of the epithelial cell characteristics—may play a role in endometriosis [58]. A hallmark of endometriosis is the dysregulation of steroid hormone production and receptors in both the eutopic and ectopic endometrium in those patients who present with the disease. This fact could also explain infertility by affecting the receptors that are necessary for embryo implantation in endometriosis [2]. Hormonal dependence is also explained by the regression of the disease in menopause or after bilateral oophorectomy [2,17]. However, the response to progesterone can be affected by the emergence of resistance, altering progesterone receptors in the ectopic and eutopic endometrium [2].

Thus, a series of factors are involved in retrograde menstruation, as we can see; for the development of lesions, the reflowed endometrial tissue must not be destroyed by the immune system, so an altered immune system is essential [59,60]. Endometrial stem cells seem to play a key role in cyclical regeneration of eutopic endometrium, but their role in endometriosis remain to be proven [61,62]. Menstrual stem cells (MenSCs) are involved in stemness and self-renewal [63]. Endometrial stem cells (EnSCs) have promising clonogenic ability in the endometrium (endometrial epithelial progenitors and stromal cells). EnSCs positively express embryonic stem cell markers such as OCT4, SOX2, and NANOG [64].

Here, we present two mechanisms (altered immune system and stem cell involvement) that seem to play a role in the development of endometriosis in more detail.

(a) Altered immune system

Macrophages could be responsible for the destruction of endometrial tissue in the peritoneum [7]. Neutrophils and phagocytic leukocytes are attracted from the circulation during the inflammatory response to the menstrual effluent, and an increase in bone-marrow-derived cells is physiologically observed before the onset of menstruation. The endometriotic environment is invaded by cells of the immune system; approximately 70–80% are macrophages, 20% are natural killer cells, and 10% are Treg cells (regulatory T cells), with a reduction in the number of dendritic cells [65].

Endometrial cells in women with endometriosis are more resistant to the cytolytic action of autologous peritoneal macrophages than in healthy controls. The failure to remove fragments of menstrual effluent from the abdominal cavity induces excessive local inflammation, with further persistent activation of macrophages [35,62]. The endometrial implants possesses an immune microenvironment comparable to that of a tumor-like inflammatory system [65,66]. Macrophages are complex cells at the center of this disease, with a critical role in the proliferation, vascularization, and innervation of lesions. Under inflammatory influence, monocytes are recruited from the blood and differentiate into macrophages in tissues where they fulfil functions such as fighting infections or repairing wounds. Moreover, in some cancers, tumor-associated macrophages (TAMs) play different roles in disease progression. Macrophages that normally maintain homeostasis can become modified such that they promote disease. The ontogeny of endometriosis-associated macrophages is poorly understood; they are derived from the endometrium along with peritoneal macrophages with a dysfunctional phenotype, recruited from the blood. Low levels of matrix metalloproteinase 9 (MMP-9) are linked to macrophages’ phagocytic activity [67,68,69,70]. The remarkable phenotypic plasticity of macrophages makes them a promising therapeutic target. In both endometriosis and cancer, macrophages guard the lesions from immune surveillance while promoting pathological cell growth, invasion, and metastasis. The specific roles of endometrial macrophages have been associated with their phagocytic role and abundant secretion of MMPs, which digest the extracellular matrix, thereby facilitating detachment during menstruation. In endometriosis, their phenotypes remain understudied [71]. Abnormal endometrial cells can induce anti-inflammatory M2 polarization of macrophages located in the peritoneal fluid. M2 macrophages can modulate the adaptive immune response, scavenge cellular debris, induce tissue repair and angiogenesis, and also increase the fibrosis in lesions [65].

Inflammation associated with the secretion of sex hormones in the endometrial tissue can lead to the development of endometriosis. The production of IL6 (interleukin 6) and the activation of MAPK (mitogen-activated protein kinase) are also increased in endometrial cells [62,67,68,70]. IL6, as a result of progesterone decline, leads to tissue breakdown and bleeding during menstruation. Endometrial cells from the menstrual phase have been proven to secrete higher amounts of IL6 and enhance the self-renewal of endometrial mesenchymal stem cells (eMSCs), but the mechanisms remain unknown [72].

The balance of Th1 and Th2 helper cells shifts towards Th2 and causes disease progression. Moreover, reduced cytotoxicity through NK cells’ activity was observed in the peritoneal fluid of women with endometriosis [65], along with significantly reduced apoptosis [62]. The exposure to inflammatory mediators contributes to increased cell proliferation and increased survival, as well as malignant transformation and cancer development. Specifically targeting the inflammatory mediators could be the proper strategy for treating both cancers and endometriosis [35,73,74].

Endometriosis is associated with the aberrant expression of cell adhesion molecules, which are increased in the peritoneal fluid of women with endometriosis [59,62]. The aforementioned genetic polymorphisms, endocrine anomalies, inflammation through IL6 and IL10, the influence of the environment, and anatomical factors such as Mayer–Rokitansky–Küster–Hauser syndrome are also involved [59,60].

Several studies have shown that the activity and number of macrophages, T lymphocytes, neutrophils, and natural killer cells in the peritoneal cavity and ectopic lesions of patients with endometriosis are significantly different from those of the same cell types in healthy populations. A large number of bioactive factors (e.g., VEGF, IL6, and IL8) are produced by abnormal lymphocytes, which also induce a chronic inflammatory microenvironment in the endometriotic tissue and the peritoneal cavity [35,64,74].

All of these factors stimulate the proliferation and survival of endometrial cells. These immunological alterations could be more a consequence than a cause of endometriosis [60].

(b) The involvement of stem cells

A possible proof for the involvement of stem cells in human endometrium regeneration is derived from in vivo xenotransplantation studies. In these studies, the researchers estimated the possibility of obtaining functional endometrial tissue from human endometrial stem cells when transplanted into immunodeficient mice [55,75,76]. Hong I.S. et al mentioned that in 1978, Prianishnikov was the first who suggested the presence of local pluripotent stem cells within the endometrial tissue with a role in regenerative process of the endometrium [77]. A number of studies have sought to investigate the hypothesis of stem cell involvement in endometriosis development. The presence of stem cell markers such as OCT4 supports the concept that a population of self-renewing, undifferentiated cells play a role in the initiation, survival, and maintenance of endometriotic lesions. OCT4 is demonstrated to be involved in endometrial cell migration [78]. The role of multiple other stem cell markers has been explored, such as SOX2 and NANOG in endometriosis. But the presence of phenotypic stem cell markers is not sufficient to demonstrate a functional role of stem cells in the pathophysiology of endometriosis. In this regard, the identification of mesenchymal stem-like cells (MSCs) within endometriotic tissues was a big step in demonstrating to play a role in endometrial regeneration [52,55]. Moggio et al. [79] in 2012 isolated MSCs from ovarian and peritoneal endometriotic implants and demonstrated MSCs increased proliferation, angiogenic, invasion, and migration properties compared to MSCs from eutopic endometrium. These studies are relevant in gathering evidence related to the importance of MSCs in ectopic and eutopic endometrium self-renewal, and also emphasize that endometriotic MSCs expressing an increased invasion, proliferation, or migration properties [53,55]. However, another study found only very few BM-MSCs (0.6–9.8%) to integrate into the endometrial glands/stroma in an in vivo transplantation study. It was not possible to detect any glands consisting only of transplanted cells. Therefore, it appears that the BM-MSCs require guidance from neighboring epithelial and stromal cells to differentiate into endometrial cells [67]. Thus, these very few BM-MSC cells might not play a significant role in monthly endometrial regeneration. Furthermore, the transplanted BM-MSCs are not able to form endometrial glands surrounded by stroma de novo. However, this is a prerequisite for endometriosis. Thus, to date it remains to be proven that endometrial stem cells play a significant role in endometrial regeneration and endometriosis.

Chan et al. [37] identified a subset of epithelial and stromal cells in human ovarian endometriomas with stem cell properties self-renewal and multilineage differentiation. This is another study which might support the stem cell theory of endometriosis pathogenesis. New evidence suggests a role for retrograde menstruation of endometrial stem/progenitor cells, now that identifying markers of these cells are available [50,55,80].

More clonogenic cells have been detected in the endometrium, with the microenvironment playing a significant role in their biological properties. In this regard, mesenchymal stromal stem cells from ectopic endometrium display pronounced immunomodulatory activity. Endometriosis may originate from Müllerian and non-Müllerian stem cells, including those from the endometrial basal layer, Müllerian remnants, bone marrow, or the peritoneum. Inherited and acquired factors lead to the development of endometriotic lesions. Genetic predisposition is complex. Epigenetic mechanisms control many of the processes involved in the immunological, histological, and biological aberrations that characterize the ectopic endometrium [37,61,62].

Endometriotic stem cells exhibit distinct immune phenotypes from eutopic endometrial stem cells by upregulating the expression of Toll-like receptors, pro-inflammatory factors, angiogenic factors, and other related factors with proliferation and invasion abilities [64]. Toll-like receptors, which are a subset of pathogen recognition receptors capable of detecting microbes and viruses, could be used as biomarkers in the early stages of the disease, while also being detectable in the peripheral blood [7].

A rather complex explanation of etiopathogenesis can comprise the following cascade: menstrual reflux through the fallopian tubes into the peritoneal cavity, followed by the degradation of hemoglobin and the toxic effect of iron, with the production of ROS and the intervention of local antioxidant factors. ROS cause cellular signaling followed by the triggering of the immune response. Exceeding the physiological antioxidant reaction causes chromosomal instability with DNA damage that leads to necrobiosis and subsequent alteration of metabolism with aerobic glycolysis (the Warburg effect). Aerobic glycolysis decreases oxidative phosphorylation and protects the cell from ROS. If the process of inflammation, acidosis, DNA damage, and ROS is excessive, malignancy occurs with cancer stem cells (CSCs). This is heavily dependent on the reactions of the immune system, the localization of endometriosis, hormonal ingestions, and toxic molecules produced during metabolism [3]. Stem cells are important in the emergence and development of endometriosis, and even more so in cancer. The association between CSCs and cancer’s resistance to chemotherapy has been confirmed by studies [67,68]. This is due to their capacity to repair DNA damage through DNA repair enzymes while dividing slowly. Checkpoint proteins and free radical scavenging are involved in this process [68,81].

Stemness is a characteristic of malignancy that has been studied in the search for markers that can serve as prognostic factors or in targeted therapy. Stemness-related genes have been found, such as SOX2 (sex-determining region Y-box 2), NANOG (Nanog homeobox), and OCT4 (octamer-binding protein 4), along with the expression of the surface markers CD133+ and CD44+ and chemokine markers such as CXCR4 on cancer stem cells as stemness markers. These markers may be useful as predictive biomarkers for prognosis in endometrial cancer, or for the development of targeted treatments [82,83,84,85]. Cancer extracellular vesicles (EVs), derived from aggressive cancer cells, play an important role in the tumor microenvironment. The recipient cancer cells of cancer EVs gain stemness properties, becoming more invasive and more metastatic. Moreover, studies have confirmed activation of the TGF-β [40], NF-kB [38], and protein kinase B [37] pathways, linked to an increase in the markers related to stemness. The upregulation of TGF-β, NF-kB, and protein kinase B by EVs was also connected to stemness through altered immune cell function [86,87,88,89].

The importance of stem cells for the nascence and persistence of both malignant tumors and endometriotic tissue is commonly appreciated. Endometriotic stem cells may be the main target for the carcinogenesis of ovarian cancer [23]. It has been suggested that endometrial stem/progenitor cells are transported in retrograde menstrual efflux through retrograde menstruation [65]. In animal models, stemness surface markers in endometriosis have been demonstrated. In a murine model of endometriosis, the role of epigenetic regulation of the Wnt/β-catenin/CD44 signaling pathway was found to be important in the invasion and migration of endometrial epithelial cells in ectopic tissue [90].

Expression of the stemness marker OCT4 was also confirmed to be increased in the epithelial cells of endometriotic lesions compared to the eutopic endometrium in women with endometriosis. As highlighted above, another transcription factor found in cancer, SOX2, was also found to be highly increased in the stromal component of ectopic progenitor cells inside endometriosis implants. Other markers of cell stemness, including CD117, CD133, ALDH1, NOTCH, and NUMB, were found to be upregulated in endometriomas. Mesenchymal stem cells (MSCs) from women with endometriosis showed increased proliferative and invasive activity compared to MSCs from healthy women [23]. Yong Song et al. [91] focused on stemness-related genes such as the transcription pluripotency factors OCT4 (octamer-binding protein 4), SOX2 (sex-determining region Y-box 2), and NANOG (Nanog homeobox) in women with endometriosis. These genes play a crucial role in the regulation of self-renewal and pluripotency in embryonic stem cells and primordial germ cells. These genes were significantly increased in endometriotic women in comparison with the control group. SOX2 mRNA and protein expression was significantly higher in the eutopic endometrium of participants in the endometriosis group, supporting a stem-cell origin of endometriosis [91]. Several other reports have also demonstrated that EnSCs positively express embryonic stem cell markers such as OCT4, SOX2, and NANOG [92].

Di Claudio, in his thesis about endometriosis, also showed the possibility of identifying potentially ‘high-risk’ patients who present a significant upregulation of genes involved in reprogramming (e.g., SOX2, NANOG), cancer metabolism (e.g., TP53, KRAS), and epithelial–mesenchymal transition (e.g., TGF alpha and SNA11). Furthermore, in 3D spheroid cultures of endometriosis, an increased co-expression of the CSC surface markers CD44 and CD133 was observed, with increased tumor invasion in the ‘high-risk’ group. These results emphasize the linkage to the malignant transformation of endometriosis, as well as providing useful information relevant to endometriosis-associated pathogenesis [93].

MenSCs (menstrual stem cells) have gained prominence in the endometriosis scientific community, given their multifunctional roles in regenerative medicine as a non-invasive source for future clinical applications. MenSCs play a role in modulating proliferation, angiogenesis, differentiation, stemness, and self-renewal [63]. A chronic inflammatory endometrial microenvironment can modulate MenSCs, providing opportunities for the etiopathogenesis of endometriosis. Regenerative medicine can also be applied in endometriosis, creating new opportunities for the development of new therapeutic strategies. We highlight the importance of the local microenvironment as a driver of malignant transformation, in addition to mutations in key cancer-driving genes [63].

Every protein or molecule associated with this pathogenic process can be used as a biomarker and targeted in a future therapeutic approach. It is interesting to determine how predictive they are for malignant transformation and how we can find similarities in the behavior of endometriosis and cancer that will lead to a shared personalized therapy.

## 3. Similarities with Cancer

Endometriosis is similar to cancer in many respects. Endometriosis can present characteristics of proliferation, invasion, metastasis, and angiogenesis, but is limited in time and space, unlike cancer [74]. Here, we present the similarities, from staging and diagnosis to treatment, to emphasize the efforts of researchers to study the applicability of targeted treatments in both pathologies, starting from the molecular bases of both.

Both pathologies have estrogen dependence, the possibility of invasion or metastasis, and the potential for recurrence [1]. Endometriosis is divided into four stages based on the amount, severity, location, depth, and size of the lesions: minimal (I), mild (II), moderate (III), and severe (IV) disease, quite similar to the staging of cancers [1]. Infiltration over 5 mm in the peritoneum is defined as deep endometriosis [1,21]. Unlike cancer staging, this staging does not predict the clinical outcome or the severity of the symptoms, so multiple classifications are necessary, as the problems of diagnosis, treatment, and follow-up have not yet been solved. Lesions usually occur in the ovaries, peritoneal cavity, abdominal scars, bladder, ureter, and bowel, and rarely in the brain, eyes, or lungs [1].

There are also similarities in terms of symptoms from the location of the pathologies or from distant metastases. From an imaging point of view, ultrasound, MRI (magnetic resonance imaging), and CT (computed tomography) are helpful. The use of some common biomarkers has also been attempted [13,14,94,95,96].

Various hypotheses regarding the pathogenesis of these two quite similar pathologies have been evaluated, in addition to EMT (epithelial–mesenchymal transition), including the involvement of growth factors, hormones, oxidative stress regulators, antibodies, pain regulators, neovascularization/angiogenesis factors, inflammation regulators, cell survival regulators, and adhesion and migration regulators [2,3,13,14,16,74,97,98,99]. Collective cell migration and epithelial–mesenchymal transition (EMT) may be closely related to the pathogenesis of deep endometriosis and cancers; the specific mechanism remains unclear [1]. In addition, in both pathologies, EMT is a mechanism that helps in the development of lesions from a stem-like cell population with a similar origin to early endometrial cells under the influence of FOXC2 (forkhead box family protein 2) and confers resistance to chemotherapy [100,101,102,103,104]. A mapping of the disease can be performed from the samples, but the origin of the disease remains unclear. The Warburg effect in etiopathogenesis, described above, can, to some extent, explain the occurrence of endometriosis and possible malignancy, while also describing the relatively malignant behavior of endometriosis and the link between these pathologies [3]. The malignant transformation of endometriosis has increased from 1/100 to 2/100, i.e., it has doubled, and according to certain statistics it has even increased fourfold [3,16].

Genetics are also involved, including translocation deletions and point mutations highlighted by the FISH test [3,14]. ARID1A (AT-Rich Interaction Domain 1A) mutations are particularly prevalent in gynecological cancers and premalignant gynecological lesions, especially those of endometrioid origin. The loss of functional ARID1A protein may induce DNA damage and decrease the cells’ ability to repair it. In ovarian and endometrial cancers, ARID1A mutations often co-occur with alterations in the PI3K/AKT (phosphoinositide 3-kinase/activated protein kinase) pathway genes KRAS (Kirsten rat sarcoma virus), PIK3CA, and PTEN (phosphatase and tensin homolog) [8,13,15,94,96,102,104,105]. The ARID1A/PIK3K/AKT pathway in endometriosis and ovarian cancer regulates the proliferation and survival of endometriotic and cancer cells [23,33]. Other genes implicated include PTEN, KRAS, and miRNAs [23]. Aberrant genes are a link between endometriosis and ovarian cancer; multipotent stem/progenitor cells show aberrant expression of genes such as KiT, HIF2 (hypoxia-inducible factor 2), and E-cadherin, and downregulation of the tumor-suppressor genes PTEN and ARID1A [106]. The key oncogenes B-cell lymphoma 6 (BCL6) and BCL9 may play a role in endometriosis as well as in the placenta, another tissue type characterized by invasion and proliferation. In these two pathologies, we have highlighted related molecular mechanisms and underlined associated research perspectives. BCL6 and BCL9 are involved in cell proliferation, migration, invasion, stem/progenitor cell maintenance, and differentiation. BCL6 is aberrantly upregulated in preeclamptic placentas and in endometriotic lesions. Increased endometrial BCL6 and BCL9 levels are considered to be a non-invasive diagnostic marker for endometriosis [23,44,107].

Various forms of proteomics are taken into account, such as metabolomics (e.g., amino acids, small molecules, metabolism products), lipidomics, and transcriptomics (miRNA, syncDNA, circDNA), which are included in the new hypotheses related to the occurrence of the disease [2,104]. Aberrant expression of miRNAs could provide potential diagnostic and prognostic biomarkers for ovarian cancer (OC), while also being implicated in chemosensitivity and resistance. MiRNAs can regulate the action of sexual hormones. Advances in targeted therapies based on the regulation of miRNAs are being evaluated [85,102]. Furthermore, there is a positive correlation between miRNA expression in plasma and eMSCs (endometrial mesenchymal stem cells) in women with endometriosis, which means that miRNAs can be used as possible biomarkers for endometriosis [69]. The literature also shows an increased frequency of adhesion molecules (e.g., VCAM and ICAM), the presence of metalloproteinases, and other factors with effects on proliferation, metastasis, angiogenesis, resistance to apoptosis, relapse after excision, and tissue invasion, all being documented in both endometriosis and malignancies [13,16,108]. Metalloproteinases 2 and 9 seem to be markers of the aggressiveness and invasion of endometriosis, as well as the risk of cancer [100,101,102,103].

From a metabolic point of view, aerobic glycolysis is the most affected process in both endometriosis and cancer [109].

From the perspective of treatment, we can find that some similarities have been confirmed and even used clinically; for example, the ENDOGRAM classification takes into account the possible resistance to hormonal therapy due to the histologically confirmed lack of hormone receptors, similar to chemoresistance and resistance to hormonal therapy in cancers [3,96]. Regarding the resistance of endometriosis to therapy, there is a similarity with the resistance of cancers to chemotherapy, a theory based on stem cell hypotheses of the occurrence of these pathologies. Stemness is the ability of stem cells to regenerate, proliferate, and differentiate. Cancers that have stemness properties are resistant to chemotherapy, and this may also apply in the case of endometriosis; FOXC2 (forkhead box protein C2) and PGF (platelet growth factor) are involved in the process [101].

Stemness markers are correlated with the response to therapy. Stemness-related genes—notably SOX2 (sex-determining region Y-box 2), OCT4, and NANOG—are responsible for maintaining the stem-cell-like phenotype, including conserving self-renewal capacity and pluripotency. Cancer stem cells are characterized by the expression of surface stemness markers such as CD44, CD117, and CD133, demonstrating both increased tumorigenicity and reduced platinum sensitivity; these stemness markers are associated with poor overall survival in cancers [110]. The Wnt signaling pathway plays a key role in the maintenance of homeostasis and in the organization of the stemness compartments. Cyclic exposure to hormonal stimulation, as a major driver of differentiation and functional determinant, represents a critical step in disease development [43,110]. New prognostic markers are needed for identifying high-risk patients. There is a link between poor prognosis and overexpression of stemness genes, with SOX2 being significantly implicated, proving the suitability of stemness genes for use in cancer management [111]. The differential expression of a panel of genes involved in the preservation of stemness, also considered to be markers of stem cells’ presence (e.g., SOX2, OCT4), has been investigated. Moreover, SALL4-positive cells may play a role in the pathogenesis of endometriosis [112].

Different studies have attempted to identify the differences between endometrial stem cells (EnSCs) isolated from the ectopic lesions of patients with endometriosis and EnSCs isolated from the eutopic endometrium of controls. A study by Yanli Liu et al. exhibited small differences; this observation supports John Sampson’s implantation theory [64]. Endometrial mesenchymal stem cells (eMSCs) are also the progenitors of endometrial stromal fibroblasts; eMSCs can enter the vascular circulation, potentially through the Cxcl12/CXCR4 axis, and lead to hematogenous dissemination. Ectopic MSCs display stronger proliferation, migration, and angiogenesis abilities than eutopic MSCs; they also exhibit distinct immune phenotypes by upregulating the expression of Toll-like receptors, pro-inflammatory factors, angiogenic factors, and other related factors, with an increasing degree of fibrosis in ectopic lesions [43,64]. Due to their increased proliferation and auto-transplantation capabilities, MSCs are ideal for cell-based therapy; they play a key role in the occurrence of endometriosis [43].

Similarities between endometriosis and cancers are presented in Table 1 in a more structured and understandable manner. 

## 4. Future Personalized Treatment Options Have Emerged from Molecular Pathology Mechanisms

As we have shown, endometriosis behaves in many aspects like a malignant pathology. This finding paves the way toward new shared therapeutic options. Two-dimensional (2D) co-cultures and, recently more common, 3D systems based on self-organization and controlled assembly are used for the in vitro modeling of endometriosis. These models are used to investigate the development of endometriosis and therapeutic methods for its treatment [23,65,113,114].

Even if cancers share some pathogenic features with endometriosis pathways, there are fundamental differences between these diseases, starting with the severity of the malignant pathology. The main treatment consists of surgery with or without adjuvant therapy. Targeted treatment applies to a niche in the event of a lack of response to treatment. Mortality is the main concern in cases of malignancy, which is the critical distinction between these pathologies. Due to the perception that endometriosis is a non-fatal condition, there is a potential shortfall in funding for research on endometriosis. However, because this disease is an economic and social burden, this perspective needs a reevaluation. Based on the common features of these pathologies at the molecular and genetic levels [74,115,116], medications initially developed for endometrial cancer have found applications in treating endometriosis [74,116].

It is important to find individualized treatments. In some situations, hormone therapy helps, but the reverse process is also valid; due to the estrogen dependence of endometriosis and the cancers developed from it or associated with it, tamoxifen can be involved in the malignant transformation. Estrogen’s role in promoting the growth and progression of the disease has been demonstrated by several clinical studies. Progesterone receptor levels are an important modulator of progesterone resistance in endometriosis [117]. Similar to resistance to classical cancer therapy, resistance to endometriosis treatment has been described, and stemness seems to be involved [67,68]. An explanation for therapeutic resistance, beyond the alteration of the hormonal receptors, could be that the treatment only addresses the differentiated cells, not the stem cells that propagate and spread the disease. Researchers determined that ectopically placed stem cells of endometrial tissue or bone-marrow stem cells outside the uterine cavity could be involved in endometriosis [25]. Stem cells are involved in proliferation, invasion and maintaining the disease, together with the microenvironment and defective immune system. The progesterone resistance or the resistance to the common treatment for endometriosis is due to the reality that this treatment is effective on differentiated ectopic endometriotic cells, while the stem cells involved in maintaining endometriotic lesions may not be affected [25]. Further investigations are needed [67,68]. Targeting molecular pathways, we attempted to outline a management direction for the future.

Various options are being explored; for example, IL6 or its effector functions can be targeted as a novel therapeutic option in endometrial cancer [118]. Tocilizumab, a monoclonal antibody, is also under research, as are various natural antioxidants and drugs approved for the treatment of other pathologies, as well as anti PD-1 (programmed cell death protein 1), which has shown the highest response rates in vitro [96]. Poly ADP-ribose polymerase (PARP) inhibitors have shown encouraging results in platinum-resistant patients [118]. Trametinib MEK1/2* (mitogen-activated protein kinase) inhibitors have unfortunately proven to promote stemness [118]. Metformin potentially inhibits ovarian cancer (OC) stem cells. Moreover, salinomycin, an antibacterial agent, is a selective inhibitor of ovarian cancer stem cells and breast cancer stem cells. The poor bioavailability of these agents can be remediated by conjugation with CD133-targeted nanoparticles. Amlodipine, a calcium channel blocker, inhibits the growth of BC stem cells. CSC therapy with CSC vaccines was able to activate immune responses to autologous tumor antigens [118].

Pluripotent stem cells can be used for disease modeling. Their selective gene encoding was a groundbreaking discovery. Several studies have attempted to find a solution to target modified genes, including the use of viral vectors (lentiviral, adeno-associated, adenoviral, retroviral) or non-viral vectors (liposomes) that drive the therapeutic gene to the host cells [119]. Pluripotent stem cells can be isolated from endometrial biopsies; menstrual blood stem cells (MenSCs) come from bodily discharge and can be obtained easily, repeatably, and non-invasively, with no ethical concerns, low immunogenicity, low tumorigenicity, and multi-lineage differentiation capacity [109]. In comparison to BM-MSCs (bone marrow mesenchymal stem cells), several studies have shown the superiority of menstrual MSCs in many respects [120,121,122,123]. Multipotent fetal stem cells are easy to acquire from Wharton’s jelly, the placenta, amniotic fluid, or the umbilical cord, and they are not associated with ethical concerns. They can be banked for autologous utilization in later life, having higher differentiation potential compared to adult stem cells. They harbor fewer mutations, are not tumorigenic, and exhibit low immunogenicity [119].

Novel targeted treatments based on the concept of CSCs are sought, including nanoparticle-based drug delivery systems for both diseases. Additionally, fertility preservation in oncology patients after anticancer treatments has been taken into consideration. Potential methods to facilitate the design of nanoparticles will arise. Viral and non-viral vectors are used for miRNA transmission. Non-viral nanoparticles and liposomes have been used in recent years. Nanoparticle delivery systems for CSC-targeted therapeutic agents were found to be more efficient and less toxic. Moreover, multiple therapeutic agents can be incorporated into one carrier. Salinomycin’s therapeutic efficacy against CD44þ drug-resistant cells was improved when conjugated with a hyaluronic acid-based nanogel [54,68,83,124,125,126,127].

Various strategies are sought to target cancer stem cells in order to block their stemness. Cancer cells need a continuous nutritional supply. These cells use various strategies, including autophagy, to avoid cell death. This works like a protective process that allows the cell to regenerate ATP. In the event of metabolic stress and a hypoxic environment, the tumor may provide sites for the expansion of cancer stem cells and rapid tumor progression through altered aerobic glycolysis in both endometriosis and cancer. CSCs are resistant to conventional treatment and have the potential to regenerate themselves indefinitely. Promising new options in cancer therapy may be derived from this in the future [74,109,128,129,130]. Genes encoding proteins that have an antioxidant effect can also have an effect in blocking the malignant transformation of endometriosis, and they may even be able to reverse the occurrence of endometriosis. These findings require validation [131].

Specific antibodies have been investigated against CSC markers. Anti-CD44 antibodies have shown their potential against different CSCs in pre-clinical investigations. Anti-CD133, another antibody that can isolate CD133 markers on CSCs, has also been developed. Transcription factors such asHIF-1a, NF-kB, and beta-catenin have also been considered as therapeutic targets against CSCs [83].

Researchers have investigated the tumor-killing activity of cytokine-induced killer (CIK) cells against cancer stem cells (CSCs) through the recognition of stemness markers such as the Oct4 and ALDH genes [132].

To attack stemness properties, we have to understand how to induce stem-like cancer cells [83,133]:

- Hypoxia: The oxygen levels in the tumor microenvironment (TME) may regulate the stemness status of CSCs. Cells cultivated under hypoxic conditions show significantly higher proliferation and infiltration capabilities.
- Some cytokines in the TME may stimulate stemness, e.g., granulocyte-stimulating factor (G-CSF), TNF (tumor necrosis factor)-alpha, or IL6.
- Mitochondrial deoxyribonucleic acid (mtDNA) blocking.
- Sex hormone stimulation may contribute to stemness [133].
- Wnt signaling plays a critical role in multiple biological processes, including cell differentiation and proliferation, survival, migration, tumorigenesis, stemness, and chemoresistance. The CSC markers are often related to hypoxic activation of the WNT/b-catenin pathway. By inhibiting these pathways to kill the CSC population, or forcing them out of dormancy and transforming them into active cells, they became more susceptible to classical therapy [134].

Wnt/beta-catenin signaling is implicated in resistance to PARP inhibitors. In this regard, targeting Wnt/beta-catenin signaling could be the solution for overcoming therapy resistance. Such signaling is also present in endometrioid ovarian carcinomas that develop from endometriosis due to CTNNB1 mutations. This demonstrates that targeting Wnt/beta-catenin signaling may serve as a promising treatment strategy for epithelial ovarian cancer (EOC) [55,135,136,137,138,139].

Within the tumor microenvironment, which plays a pivotal role in tumor growth, tumor-associated macrophages (TAMs) profoundly influence tumor progression. TAMs promote tumor cell invasion by mediating the degradation of the extracellular matrix through the involvement of MMP-2, MMP-7, and MMP-9. In hypoxic conditions in tumors, TAMs produce various angiogenic factors with a role in metastasis. Strategies targeting TAMs represent a potential therapeutic avenue. Nanotechnology-based active targeting therapies that specifically target TAMs within the tumor microenvironment facilitate the selective accumulation of nanoparticles at target sites, bypassing off-target toxicity concerns [140]. A subset of studies has targeted the proliferation of TAMs in an effort to alleviate tumor burden and improve clinical outcomes. After targeting the Csf-1 receptor, a significant reduction in macrophage numbers and tumor size was demonstrated, along with an improvement in clinical outcomes and symptoms in patients with cancer. Csf-1 is a key regulator of macrophage proliferation [67,68,70,71]; it can also be used in endometriosis. Toll-like receptor (TLR) agonists constitute a promising group of anti-TAM drugs (for example, imiquimod). Additionally, macrophages express estrogen receptors. Tamoxifen, an anti-estrogen-receptor antagonist, appears in various anticancer regimens. Macrophage migration inhibitory factor (MIF) represents a promising target for cancer immunotherapy. STAT3 modulation is an attractive strategy for the induction of a pro-inflammatory phenotype in macrophages. Selective elimination of VEGFR1+ macrophages has shown efficacy within endometriotic lesions [71].

Another potential mechanism of therapeutic management could involve blocking the recruitment of disease-promoting macrophage populations. Phase I trials based on this approach have shown that the CLC2/CCR2 recruitment mechanism is implicated in a number of cancers. The inhibition of recruitment may be beneficial in blocking the infiltration of macrophages into endometriotic lesions; however, the mechanism of recruitment is poorly understood [70].

Targeted therapies directed against various molecular pathways are under research, such as the use of bevacizumab as a VEGF pathway inhibitor, or PARP inhibitors such as olaparib or niraparib. MEK inhibitors and PIK3K/mTOR/Akt inhibitors are currently being tested in ovarian cancer (OC); they also decrease cell proliferation and infiltration in deep endometriosis, as confirmed in murine models. Immunotherapy targeting PD1/PD-L1 expression could be a future solution in endometriosis-associated OC [105]. MiRNAs, as feasible biomarkers for the early diagnosis of endometriosis in symptomatic and non-symptomatic women, are also useful for planning targeted management protocols [141].

All-trans retinoic acid (ATRA) inhibits the EMT of endometriotic stromal stem cells. Large endometriotic stromal colony-forming units (CFUs) are sets of endometriotic stromal stem cells with high potential for self-renewal, proliferation, invasion, and EMT, along with upregulated IL6. After ATRA treatment, the expression of IL6 was significantly reduced, accompanied by a decrease in the EMT, migration, and invasion of stromal CFUs. The inhibition of EMT under ATRA treatment was mediated by IL6 [130].

In vivo administration of 3-deazaneplanocin, an EZH2 inhibitor, significantly inhibited the growth of endometriotic lesions, attenuated EMT, and reduced fibrosis and hyperalgesia. An enhancer of zeste homolog 2 (EZH2) induced EMT in both endometriosis and cancers [131,142,143]. In the case of progesterone resistance, the use of low-dose tacrolimus in treating female infertility improves embryo implantation [144]. It is used in PCOS, but both diseases are associated with sex steroid hormone receptors. Understanding PCOS and the pathological roles of endometriosis in sex steroid receptors resistance is crucial. This approach can be an avenue for developing potential therapeutic strategies to treat infertility in both conditions [145]. Also, women with PCOS are at increased risk for endometrial cancer, which is another link between these diseases [146].

## 5. Discussion

Endometriosis is an economic and healthcare burden [17], which is why there is a constant concern related to this pathology. There is still no standardized approach to the treatment of endometriosis [5], and the studies show heterogeneous data [5,11,21]. Randomized studies are needed with a properly thought-out study design (an unbiased fashion design) that respects the Standard for Reporting of Diagnostic Accuracy (START), which incorporates a checklist that must be respected [5,11,20,21]. The studies thus far—due to the use of incorrect study designs, some of them on small samples—may cause us to miss non-obvious opportunities, and some of the biomarkers that have already been investigated could be underestimated [5].

At the moment, studies are focused on targeted therapy for both endometriosis and cancer. In particular, due to the link between these pathologies, finding their common risk factors is an essential concern, in order to remove them as part of the therapy. The use of natural methods or drugs that are approved for other pathologies, as well as the discovery of new therapeutic approaches, is at the center of researchers’ concerns [13,96]. This is also due to the resistance to therapy observed in cases of both endometriosis and cancers associated with or derived from endometriosis [12,96,143,147]. We have more questions than answers. Perhaps endometrial stem cells are implicated in both endometriosis and cancer, influenced by sex hormones and other microenvironmental factors, such as ROS, which can damage DNA. The high oxidative potential of free iron in the malignant transformation of endometriosis has been emphasized by Stemp et al. [8,15]. Why do we care so much? The lack of standardized protocols and diagnostic criteria in pathology practice makes it difficult to study and classify this disorder [4].

However, we must also be aware of the differences between these pathologies, which are major. We must start with the fact that the main characteristic of malignancies is high mortality [74]. Regarding the pathogenesis of the diseases, the sequence of events is the same, but the individual steps differ significantly. Another difference is that, in endometriosis, the endometrial stroma usually migrates along with the epithelium and then integrates into the stroma of the host tissue. In cancers, altered epithelial cells migrate and integrate into the host stroma, forming metastases. Cancer metastases can significantly alter the environment in the host tissue, which, with few exceptions, represents a clear contrast to endometriotic implants, which are very limited in size [58,74]. Furthermore, the management of cancers consists of surgery and adjuvant chemotherapy and radiotherapy. Personalized treatment is a niche therapy used in the event of a lack of response to classical therapy. The severity of the prognosis justifies the efforts of researchers to find a curative treatment for cancer. Due to some common features between cancer and endometriosis, these personalized treatments could be applicable in endometriosis as well. Thus, we can look at endometriosis as a neoplastic process, as mentioned above, but limited in time and space [74,148,149].

Surgery is still the main therapy, laparoscopy and robotic surgery being the best option [150]. Regarding surgery in endometriosis patients, surgeons should be aware of the increased risk of complications during surgery for suspected endometriosis [151]. Clinical application of current knowledge would involve targeted therapy that acts on cellular/molecular aberrations to reverse the process, the discovery of the link between endometriosis and malignancy, clinical and pathological features, and prognosis. In addition, an intense concern of researchers is the creation of a genetic risk profile, risk stratification, preoperative biomarker evaluation, and the identification of gene-based biomarkers that are useful in final outcomes and follow-up [13,96,152,153]. Machine learning techniques have excellent ability to deal with complex data, showing good prospects [1,9,152,153].

Different pathogenic pathways and different behaviors of potential new markers that are uncovered should be studied. According to our findings based on the literature, stemness is the characteristic on which resistance to therapy is based, calling for efforts to find molecular pathways that can be targeted by personalized treatment. Each of these patients would need this treatment once a month until the menopause; this would be a burden. More research is needed before we can draw firm conclusions.

## 6. Conclusions

Endometriosis is a disease with a complex pathogenesis that shares numerous features with cancers, but it also has important differences. In this review, we have tried to highlight common pathways in cancer and endometriosis with clinical applicability, with the caveat that, unlike endometriosis, cancer is a disease with fatal evolution, whose conventional treatment is based on chemo/radiotherapy. Here, we focus on the niche of common pathogenic features and personalized treatments that target molecular pathways. Our findings show that, in both pathologies, the resistance to treatments is due to stemness characteristics, with stem cells playing a key role in the appearance and evolution of both diseases. Thus far, we have no satisfactory answer for these issues that would be acceptable to the scientific community.

## Figures and Tables

**Table 1:** Common behavioral features in endometriosis and cancer.

1. Etiopathogenic Mechanisms [2,3,13,14,15,16,17]		- Response to TGF and IGF growth factors - Proliferation - Apoptosis resistance - Angiogenesis involving VEGF - Invasion involved decreases in MMP-2 and MMP-9, the adhesion molecules ICAM and VCAM, and E-cadherin - Metastasis, the same factors involved as in invasion - Genomic instability - Estrogen addiction - Genetic polymorphism in the production of estrogen and progesterone receptors in endometriotic tissue/cancer - EMT - Stem cells/stem cell markers - Involved environmental factors, e.g., exposure to dioxin - Involved inflammatory mediators, e.g., ILs, adhesion molecules ICAM and VCAM, TNF-α - Epigenetic factors; aberrant hypermethylation followed by chromosomal aberrations (DNA damage) - Genetic: deletions, translocations, mutations, loss of reparative enzymes - Oxidative stress - Hemorrhage in endometriomas or neoplastic tumors with the release of iron ions, causing oxidative stress - Crosstalk between ectopic and eutopic endometrium - Omics: metabolomics, lipidomics, proteomics, transcriptomics (miRNAs, circRNAs)			
2. Diagnostic Methods [1,3,5,6,8,9,10,13,14,15,16,18,19,20,21,23,54,55,59,94,95,96,108]		Clinical	Similar symptoms Associated comorbidities: autoimmune disease (lupus erythematosus, rheumatoid arthritis), cardiovascular disease (HT), chronic fatigue		
		Imaging	- Ultrasound, CT, MRI		
		Laparoscopy with Samples Laparotomy	- Gold standard		
		Biomarkers (under study: not validated for endometriosis; some validated for cancer)	- VCAM and ICAM - Growth factors, e.g., IGF, TGF - Tumor markers, e.g., CA125 - Inflammatory markers, e.g., IL6, IL8, NLRP3 - VEGF - Hormonal markers - Matrix-related molecule (osteopontin) - Markers of apoptosis - Oxidative stress markers; free iron’s role in malignancy - Free circulating DNA; DNA repair - Omics: miRNAs, circRNAs - Genetic expression candidate biomarker screening - Different binding proteins - Immune cell subtypes		
3. Staging [1,21]		In endometriosis: rASRM in minimal (I), mild (II), moderate (III), severe (IV) disease.	In cancer: FIGO/TNM		
4. Metastases or Recurrences [2,5,6,11,20,96,101]		- In endometriosis and cancers, metastases in the ovaries, peritoneum, intestines, urinary tract, and abdominal scars; seldom in the brain or eyes			
5. Disease Management [2,5,6,11,13,16,20,21,22,25,96,101,108]	Current Management ▪ Surgical ▪ Hormonal ▪ Pain relief	Treatments First Administered in Cancer, Currently also Used in Endometriosis/Adenomyosis: GNRH agonists and antagonists, progesterone	Future Therapy: ▪ sFlt1 as human anti-VEGF A ▪ Adenovirus as an angiostatin vector inhibits angiogenesis ▪ Targets under evaluation: aromatase inhibitors, COX2 inhibitors, anti-VEGF bevacizumab ▪ Anti-IL6, tocilizumab, other monoclonal antibodies ▪ Antioxidants, anti-PD-1; ▪ Genetically guided therapy ▪ Doxorubicin ▪ Reversal of the EMT process	Resistance to Therapy - By their stemness	Management of Fertility: - ART, oocyte vitrification in cancer and endometriosis, oncofertility
6. Prognosis/Follow-up [2,5,13,14,15]		▪ It is necessary to find a risk stratification for the development of cancer from endometriosis			
7. Common Risk Factors [13,14,15]		▪ Genetic and environmental factors such as lifestyle, smoking, physical activities, caffeine intake, ethnicity (more frequently Asians), estrogen intake, BMI, insulin resistance, inflammation, oxidative stress, diabetes, and hypertension ▪ Protective factors: progesterone, breastfeeding			
8. The Most Distinct Metabolic Alteration in Both Diseases [109]		▪ Altered aerobic glycolysis			

TGF—transforming growth factor, IGF—insulin-like growth factor, VEGF—vascular endothelial growth factor, MMP-2—matrix metalloproteinase 2, MMP-9—matrix metalloproteinase 9, ICAM—intercellular adhesion molecule, VCAM—vascular cell adhesion molecule, EMT—epithelial-to-mesenchymal transition, IL—interleukin, TNF-α—tumor necrosis factor alpha, miRNA—microRNA, circRNA—circular RNA, HT—hypertension, CT—computed tomography, MRI—magnetic resonance imaging, CA125—cancer antigen 125, IL6—interleukin 6, IL8—interleukin 8, NLRP3—nucleotide-binding domain, leucine-rich-containing family, pyrin domain-containing-3, rASRM—revised American Society for Reproductive Medicine, FIGO—International Federation of Gynecology and Obstetrics, TNM—classification of malignant tumor, sFlt1—soluble fms-like tyrosine kinase-1, COX2—cyclooxygenase-2, anti-PD-1—anti–programmed death-1, ART—assisted reproductive technology, GNRH—gonadotropin-releasing hormone, BMI—body mass index.
